# Perioperative management of non-metastatic gastroesophageal cancer in a Philippine university hospital: a 10-year experience

**DOI:** 10.1186/s12957-025-04190-6

**Published:** 2026-02-04

**Authors:** Dawn Andrea N. Fontanar, Shiela S. Macalindong

**Affiliations:** Department of Surgery, University of the Philippines – Philippine General Hospital, Manila, Philippines

**Keywords:** Gastroesophageal junction cancer, Perioperative management, Surgical oncology, Resource-limited setting, Survival

## Abstract

**Background:**

Gastroesophageal junction (GEJ) cancer has remained a significant global health challenge due to late-stage diagnosis and poor survival outcomes. This study presents the 10-year institutional experience in the multimodal management of non-metastatic GEJ cancer in a low-resource setting.

**Methods:**

This is a retrospective cohort study of 101 patients with non-metastatic GEJ cancer. Data on demographics, tumor characteristics, management approach, and outcomes were analyzed. Outcomes of patients who underwent neoadjuvant therapy versus outright surgery were compared.

**Results:**

Majority of the patients were males (76%), with a mean age of 56 years old (SD 11.6) and adenocarcinoma as the predominant histology (85%). Of the cohort, 40% underwent neoadjuvant therapy, predominantly FLOT chemotherapy regimen (37%) and CROSS chemoradiotherapy regimen (54%). Definitive surgery primarily left thoracoabdominal approach with distal esophagectomy and total gastrectomy, and Roux-en-y esophagojejunostomy was performed in 60% of the cases. Neoadjuvant therapy was associated with reduced margin positivity and improved tumor regression; however, it had a high rate of incomplete treatment due to toxicity or progression. Surgical and medical complications occurred in 18% and 26%, respectively, with a 7% in-hospital mortality rate. The overall survival was 14%, and the 2- and 5-year cancer-specific survival was higher in the neoadjuvant group compared to upfront surgery.

**Conclusions:**

This study underscores the benefits of a multimodal treatment strategy in non-metastatic GEJ cancer; however, challenges, including low institutional surgical volumes and treatment-related toxicity, highlight areas for improvement. Strengthening multidisciplinary collaboration and access to advanced systemic therapies are essential to optimize patient outcomes in resource-limited settings.

## Introduction

Esophageal cancer is the 8th most commonly diagnosed cancer and the 6th leading cause of death worldwide, with age-standardized rate of 6.3 per 100,000 population as of 2020 [1]. Gastroesophageal (GEJ) cancers have the epicenter within the proximal and distal 5 centimeters (cm) of the cardia and majority of neoplasms arising from this location are adenocarcinomas [2]. Data from the SEER program highlights that majority of GEJ adenocarcinoma were diagnosed at locally advanced or metastatic stage with five-year overall survival of 12% and 2%, respectively [3]. Management of GEJ adenocarcinomas relies on multimodal approach given the anatomic location. Surgery is still a major component of treatment and with improvement in staging techniques and post-surgical care, surgical morbidity and mortality has declined in the recent years. In addition, preoperative chemoradiation and perioperative chemotherapy have been shown to significantly improve overall survival in resectable, locoregionally advanced EGJ cancers in several randomized controlled trials [4, 5]. 

In a ten-year review of GEJ adenocarcinoma in the Philippines, majority were locally advanced when diagnosed with in-hospital morbidity of 40%, mortality rate of 4.5%, and 1-year disease-free survival rate of 69.4%. Notably, only one out 88 patients (1.1%) received neoadjuvant therapy despite majority being locoregionally advanced. The study emphasized the importance of multimodal approach to improve outcomes [6]. 

Since the previous study, there has been increased utilization of neoadjuvant therapy in recognition of the poor outcomes and in adherence to international standards of care. This study thus aims to assess the treatment outcomes of non-metastatic resectable GEJ carcinoma who underwent perioperative therapy.

## Objectives

### General objective

To present the 10-year institutional experience of the University of the Philippines - Philippine General Hospital (UP-PGH) in the multimodal treatment approach in the management of non-metastatic gastroesophageal (GEJ) carcinoma and its outcomes.

### Specific objectives

1. To determine the clinicodemographic profile of patients with non-metastatic GEJ carcinoma from January 1, 2014 to December 31, 2023.
2. To identify the preoperative workup performed to evaluate patients with non-metastatic GEJ carcinoma.
3. To characterize the GEJ cancers according to histology, Siewert classification and preoperative staging (American Joint Committee on Cancer 8th edition).
4. To identify the rate of utilization of neoadjuvant therapy and the type of neoadjuvant therapy given.
5. To determine extent of completion of planned preoperative therapy of GEJ cancer patients, including presence of morbidities during preoperative treatment.
6. To enumerate the surgical procedures performed for patients with GEJ cancer according to intent (definitive – curative vs. palliative, non-definitive), approach, resection type, and reconstruction procedure.
7. To identify the final histopathologic results in terms of margin status, nodal harvest, pathologic high-risk features, and treatment effect.
8. To identify the in-hospital and 30-day outcomes of GEJ cancer patients who underwent surgery.
9. To identify the adjuvant therapy given to GEJ cancer patients following surgery.
10. To compare the clinicodemographic, pathologic and treatment factors between outright definitive surgery and neoadjuvant therapy.
11. To determine the long-term outcomes following preoperative therapy and surgery.
12. To compare the overall survival (OS) and cancer-specific survival (CSS) rates at 1, 2 and 5 years between outright surgery and neoadjuvant therapy.

## Methods

### Research design

This was a retrospective cohort analysis conducted through chart review.

### Sampling design and sample size

All patients diagnosed with nonmetastatic gastroesophageal cancer surgically managed in UP-PGH from January 1, 2014 to December 31, 2023 who satisfied the inclusion criteria were included in this study. Total enumeration of all cases satisfying the set criteria was performed.

### Data collection procedure and analysis plan

Eligible patients were identified using the Integrated Surgical Information System (ISIS) surgical database and conference reports. From the database, search terms “gastroesophageal cancer”, “esophagogastric cancer”, “GEJ cancer”, “EGJ cancer”, “gastroesophageal mass”, “espophagogastric mass” under the dates of January 1, 2014 to December 31, 2023 were used to screen patients for inclusion. After the selection of appropriate cases, review of in-patient and outpatient medical records, operative reports and histopathology reports were done. Data collected included age, gender, patient comorbidities, performance status, nutritional risk assessment, serum albumin level, body mass index (BMI), chief complaint, preoperative workup performed, preoperative biopsy result, preoperative stage, neoadjuvant and adjuvant therapy given, surgical procedure (definitive or palliative), surgeon type (trainee or AS), histopathology results (final histopathology, margin status, lymph node harvest, response to neoadjuvant therapy and pathologic stage), immediate postoperative complications (anastomotic leak, chyle leak, surgical site infection and medical complications), repeat surgery performed, length of hospital stay, discharge status, re-admission, and presence of locoregional and distant recurrence on follow-up. A letter of request was submitted to the Philippine Statistics Authority (PSA) and a non-disclosure agreement was signed between the PSA and the investigator and co-investigator to access the death certificates of patients who died, including the date of death and the immediate cause of death (COD).

### Inclusion and exclusion criteria

All patients diagnosed with nonmetastatic gastroesophageal who satisfied the inclusion criteria were included in this study. Patients with metastatic disease, nonmalignant causes of esophagogastric obstruction, those who did not undergo any surgical procedure and those with incomplete medical records were excluded.

### Statistical analysis

Clinicodemographic, pathologic and treatment factors and outcome data were analyzed using descriptive statistics, with means and standard deviation for continuous variables and frequencies with percentages for categorical variables. Chi-square test was used to compare the clinicodemographic profile, treatment factors and outcomes between outright surgery and perioperative therapy. Long-term outcome measures, including 1-year, 2-year and 5-year overall survival (OS), cancer-specific survival (CSS) and recurrence rates were evaluated and compared between treatment modality. SPSS (version 29.0.2) was used for all statistical analyses and the level of significance was set at p-value $\:\le\:$ 0.05.

## Results

### Clinicopathologic profile

There were 101 patients included in the study and majority were males with mean age of 56 years old. Majority had normal BMI, with mean serum albumin at 34 g/L (SD 6.8).

The most common presenting symptom was dysphagia (70, 69%), and all of the patients underwent esophagogastroduodenoscopy with biopsy and contrast-enhanced CT scan of chest and abdomen for preoperative workup. Adenocarcinoma was the most common histopathologic diagnosis (86, 85%). Majority of the patients who underwent outright definitive surgery were Siewert III, while those who underwent neoadjuvant therapy prior to surgery were Siewert II. For preoperative staging, majority of the patients in all groups were cT3N1, however, the patients who underwent outright definitive surgery were at least cT2N0, while those who underwent neoadjuvant therapy had at least cT3N0 tumors.

There was no significant difference between the groups in comorbidities, nutritional status, presenting symptoms and histologic diagnosis, but there was significant difference among the groups in age, Siewert classification and clinical stage. (Table 1)

**Table 1:** Clinical profile of non-metastatic GEJ carcinoma admitted and surgically managed from 2014 to 2023

Mean (SD) / Frequency (%)	Overall N = 101	Outright Definitive Surgery N = 43	Neoadjuvant Treatment	Palliative Surgery N = 40	*p* value
			Definitive Surgery N = 18		
Age (years)	56 (SD 11.6)	56 (SD 10.8)	50 (SD 13.2)	59 (SD 10.8)	.031
Gender					.904
Males	77 (76%)	33 (77%)	13 (72%)	31 (78%)	
Females	24 (24%)	10 (23%)	5 (28%)	9 (22%)	
Comorbidities					.312
None	61 (61%)	27 (63%)	12 (67%)	22 (55%)	
Hypertension	13 (13%)	4 (9%)	2 (11%)	7 (18%)	
Diabetes mellitus	3 (3%)	1 (2%)	1 (5%)	1 (3%)	
Heart disease	0	0	0	0	
Pulmonary disease	6 (6%)	2 (5%)	3 (17%)	1 (3%)	
Multiple comorbidities	18 (18%)	9 (21%)	0	9 (22%)	
Nutritional status					
Body mass index					.280
Underweight	11 (11%)	3 (7%)	3 (17%)	5 (12%)	
Normal	20 (20%)	6 (14%)	5 (28%)	9 (22%)	
Overweight	3 (3%)	2 (5%)	1 (5%)	0	
Obese	4 (4%)	1 (2%)	2 (11%)	1 (3%)	
Not specified	63 (62%)	31 (72%)	7 (39%)	25 (63%)	
Serum albumin level (g/L)	34.3 (SD 6.8)	33.9 (SD 6.5)	34.7 (SD 9)	34.5 (SD 5.9)	.072
Nutritional risk assessment					.107
Low risk	0	0	0	0	
Moderate risk	9 (9%)	4 (9%)	2 (11%)	3 (7%)	
High risk	9 (9%)	0	3 (17%)	6 (15%)	
Not done	83 (82%)	39 (91%)	13 (72%)	31 (78%)	
Presenting symptom					.881
Dysphagia	70 (69%)	31 (72%)	12 (68%)	27 (68%)	
UGIB	6 (6%)	1 (2%)	1 (5%)	4 (10%)	
Abdominal pain	10 (10%)	5 (12%)	1 (5%)	4 (10%)	
Vomiting	10 (10%)	4 (9%)	3 (17%)	3 (7%)	
Early satiety	5 (5%)	2 (5%)	1 (5%)	2 (5%)	
Incidental finding	0	0	0	0	
Others	0	0	0	0	
Preoperative workup done					
EGD with biopsy	101 (100%)	43 (100%)	18 (100%)	40 (100%)	
CT scan	101 (100%)	43 (100%)	18 (100%)	40 (100%)	
Bronchoscopy	2 (2%)	1 (2%)	0	1 (3%)	
Pulmonary Function Test	34 (34%)	15 (35%)	7 (39%)	12	
PET-CT scan	0	0	0	0	
Others	0	0	0	0	
Histopathologic diagnosis					.086
Adenocarcinoma	86 (85%)	37 (86%)	18 (100%)	31 (78%)	
Squamous cell carcinoma	6 (6%)	3 (7%)	0	3 (7%)	
Other	2 (2%)	2 (5%)	0	0	
No biopsy done	7 (7%)	1 (2%)	0	6 (15%)	
Siewert classification					.047
Siewert I	11 (11%)	3 (7%)	0	8 (20%)	
Siewert II	27 (27%)	11 (26%)	9 (50%)	7 (18%)	
Siewert III	28 (28%)	12 (28%)	7 (39%)	9 (22%)	
Not classified	35 (34%)	17 (39%)	2 (11%)	16 (40%)	
Preoperative tumor status					.009
T1	0	0	0	0	
T2	8 (8%)	8 (19%)	0	0	
T3	36 (36%)	14 (32%)	8 (45%)	14 (35%)	
T4a	18 (18%)	6 (14%)	6 (34%)	6 (15%)	
T4b	2 (2%)	1 (2%)	1 (5%)	0	
Unknown	37 (36%)	14 (33%)	3 (16%)	20 (50%)	
Preoperative nodal status					.011
N0	14 (14%)	8 (18%)	2 (11%)	3 (7%)	
N1	37 (36%)	18 (42%)	8 (45%)	11 (28%)	
N2	9 (9%)	1 (2%)	2 (11%)	6 (15%)	
N3	5 (5%)	2 (5%)	3 (16%)	0	
Unknown	36 (36%)	14 (33%)	3 (16%)	20 (50%)	

### Surgical intervention

60% of the patients underwent definitive surgical intervention, with majority (43, 42%) had outright surgery. Majority of surgical approach was left thoracoabdominal (37, 61%) distal esophagectomy with total gastrectomy (37, 61%) and the most common surgical reconstruction was Roux-en-y esophagojejunostomy (40, 66%).(Table 2).

**Table 2:** Definite surgical procedure performed of patients diagnosed with GEJ carcinoma

Mean (SD) / Frequency (%)	Overall N = 61	Outright Definitive Surgery N = 43	Post-neoadjuvant therapy N = 18	*P* value
Surgical approach				
OPEN				.474
• Laparotomy	13 (21%)	9 (21%)	4 (22%)	
• Left thoracoabdominal (Akiyama)	37 (61%)	27 (63%)	10 (56%)	
• Transhiatal	10 (16%)	7 (16%)	3 (17%)	
• Three-field (McKweon)	1 (2%)	0	1 (5%)	
• Thoracotomy and laparotomy (Ivor-Lewis)	0	0	0	
TOTAL MINIMALLY INVASIVE APRROACH	0	0	0	
LAPRASCOPIC-ASSISTED	0	0	0	
Extent of surgical resection	8 (13%)	7 (16%)	1 (5%)	.787
• Distal esophagectomy with proximal gastrectomy	37 (61%)	25 (59%)	12 (67%)	
• Distal esophagectomy with total gastrectomy	5 (8%)	3 (7%)	2 (11%)	
• Total esophagectomy with proximal gastrectomy	8 (13%)	6 (14%)	2 (11%)	
• Total esophagectomy with total gastrectomy	2 (3%)	1 (2%)	1 (5%)	
• Total thoracic esophagectomy	1 (2%)	1 (2%)	0	
• Total gastrectomy				
Surgical reconstruction	5 (8%)	4 (9%)	1 (5%)	
• Esophagogastrostomy (thoracic anastomosis)	7 (11%)	4 (9%)	3 (17%)	
• Gastric pull-up (cervical anastomosis)	40 (66%)	28 (66%)	12 (67%)	.858
• Roux-en y esophagojejunostomy	8 (13%)	6 (14%)	2 (11%)	
• Colonic interposition	1 (2%)	1 (2%)	0	
• vDouble tract reconstruction				

On the other hand, 40% of the patients underwent palliative surgical interventions only. There were two who underwent outright surgical bypass, one gastric pullup and the other, colonic interposition. Feeding tubes were inserted on majority of these patients, 85% feeding tube jejunostomy and 15% feeding gastrostomy tube. (Table 3)

**Table 3:** Non-definite surgical procedure performed on patients diagnosed with GEJ carcinoma

Mean (SD) / Frequency (%)	Overall N = 40	Outright Non-definitive surgery N = 21	Post-neoadjuvant therapy N = 19
Non-definitive procedure / Palliative surgery only			
• Surgical bypass for obstruction	2 (5%)	2 (10%)	0
• Tube jejunostomy	34 (85%)	17 (80%)	17 (89%)
• Tube gastrostomy	4 (10%)	2 (10%)	2 (11%)

### Histopathology results

Majority (57, 93%) of the patients who underwent definite resection had adenocarcinoma as the final histopathologic diagnosis. Proximal resection margins was positive in 10% of patients overall, higher in outright definitive surgery than those in post-neoadjuvant treatment (12% vs. 6%, *p 0.468*), while distal resection margin was positive in 3%, higher in post-neoadjuvant therapy (2% vs. 6%, *p 0.518*) The mean nodal harvest was 15.5 (SD 10.9) lymph nodes overall, with higher mean nodal harvest in the neoadjuvant group than the outright surgery (14.7 ± 9.7 vs. 17.6 ± 13.5, *p 0.342*).

Among patients who underwent neoadjuvant treatment, majority of tumor regression grade was not assessed. However, there was one patient who had complete pathologic response (pCR 6%), with 12% of patients having partial response and 27% with no response to neoadjuvant treatment. (Table 4)

**Table 4:** Histopathologic results of patients with GEJ carcinoma who underwent definitive surgery

Mean (SD) / Frequency (%)	Overall N = 61	Outright Definitive Surgery N = 43	Post-neoadjuvant therapy N = 18	*P* value
Histopathologic diagnosis				.302
Adenocarcinoma	57 (93%)	40 (93%)	17 (94%)	
Squamousntd cell carcinoma	2 (3%)	2 (5%)	0	
GIST	1 (2%)	1 (2%)	0	
No tumor seen	1 (2%)	0	1 (6%)	
Margin status				
Proximal margin				.468
Positive	6 (10%)	5 (12%)	1 (6%)	
Negative	55 (90%)	38 (88%)	17 (94%)	
Distal margin				.518
Positive	2 (3%)	1 (2%)	1 (6%)	
Negative	59 (97%)	42 (98%)	17 (94%)	
Nodal harvest				
Total harvest, mean (SD)	15.5 (SD 10.9)	14.7 (SD 9.7)	17.6 (SD 13.5)	.342
Positive nodal harvest, mean (SD)	4 (SD 5.9)	4.5 (SD 6.1)	3 (SD 5.4)	.375
Lymphovascular involvement	31 (51%)	22 (51%)	9 (50%)	.307
Histologic grade				
Grade I	27 (44%)	20 (47%)	7 (39%)	.121
Grade II	14 (23%)	7 (16%)	7 (39%)	
Grade III	15 (25%)	13 (30%)	2 (11%)	
Not specified	5 (8%)	3 (7%)	2 (11%)	
Treatment effect (Tumor Regression Grade)				
Grade 0 (complete response)	-	-	1 (6%)	
Grade 1 (near-complete response)	-	-	1 (6%)	
Grade 2 (partial response)	-	-	1 (6%)	
Grade 3 (no response)	-	-	5 (27%)	
Not specified	-	-	10 (55%)	
Pathologic tumor status				.398
T0	1 (2%)	0	1 (6%)	
T1	0	0	0	
T2	8 (13%)	5 (12%)	3 (17%)	
T3	23 (38%)	19 (44%)	4 (22%)	
T4a	6 (10%)	4 (9%)	2 (11%)	
T4b	4 (6%)	2 (5%)	2 (11%)	
Not specified	19 (31%)	13 (30%)	6 (33%)	
Pathologic nodal status				.655
N0	11 (18%)	6 (14%)	5 (27%)	
N1	12 (20%)	10 (23%)	2 (11%)	
N2	11 (18%)	8 (19%)	3 (17%)	
N3	8 (13%)	6 (14%)	2 (11%)	
Not specified	19 (31%)	13 (30%)	6 (33%)	
Final Siewert Classification				.360
Siewert I	1 (2%)	1 (2%)	0	
Siewert II	30 (49%)	20 (47%)	10 (55%)	
Siewert III	26 (43%)	19 (44%)	7 (39%)	
Not specified	4 (6%)	3 (7%)	1 (6%)	

### Neoadjuvant therapy

There were 37 (40%) patients who underwent neoadjuvant therapy, 65% who underwent neoadjuvant chemotherapy, while 35% underwent chemoradiotherapy. FLOT (fluorouracil, leucovorin, oxaliplatin and docetaxel) was the most common regimen used with 67% completion. Majority of those who did not complete the neoadjuvant chemotherapy had disease progression and had one treatment mortality. Notably, there was one patient who had a complete pathologic response (pCR) with the FLOT regimen. 54% of those who underwent neoadjuvant chemoRT were given the CROSS regimen (carboplatin with paclitaxel-based chemoradiotherapy) with 92% completion.(Tables 5 and 6).

**Table 5:** Neoadjuvant therapy and treatment outcomes for patients with GEJ adenocarcinoma

Mean (SD) / Frequency (%)	Overall N = 37	Completed (N = 28)		Not Completed (N = 9)				
		Surgery		Surgery		Causes		
		Definitive Surgery N = 17	Palliative Surgery N = 11	Definitive Surgery N = 1	Palliative Surgery N = 8	Progression N = 7	Adverse Effects N = 1	Expired N = 1
Neoadjuvant	N = 24							
Chemotherapy								
Capecitabine	2 (8%)	1	0	0	1	1	0	0
ECF	4 (17%)	3	1	0	0	0	0	0
EOX	2 (8%)	2	0	0	0	0	0	0
FLOT	9 (37%)	6	1	1	1	1	0	1
FOLFOX	3 (13%)	0	1	0	2	2	0	0
Not specified	4 (17%)	0	1	0	3	2	1	0
Neoadjuvant	N = 13							
chemoRT								
CROSS	7 (54%)	3	4	0	0	0	0	0
FU with RT	2 (15%)	2	0	0	0	0	0	0
FLOT with RT	1 (8%)	0	1	0	0	0	0	0
Cisplatin, FU + RT	1 (8%)	0	1	0	0	0	0	0
Not specified	2 (15%)	0	1	0	1	1	0	0

**Table 6:** Neoadjuvant therapy and pathologic response assessment of patients who underwent definitive surgery

Mean (SD) / Frequency (%)	Overall N = 18	Grade 0 N = 1	Grade 1 N = 1	Grade 2 N = 1	Grade 3 N = 5	Not specified N = 10
Neoadjuvant chemotherapy						
Capecitabine	1 (5%)	0	0	0	1	0
ECF	3 (17%)	0	0	0	0	3
EOX	2 (11%)	0	0	0	0	2
FLOT	7 (39%)	1	1	0	3	2
Neoadjuvant chemoradiotherapy						
CROSS	3 (17%)	0	0	0	0	3
FU with RT	2 (11%)	0	0	1	1	0

### Postoperative complications

Among those underwent definitive surgery, 18% developed surgical complications, while 26% had medical complications. The anastomotic leak rate was 10%, higher in the neoadjuvant group than in the outright surgery (9% vs. 11%, *p 0.829*). There was one chyle leak on the neoadjuvant group. Surgical site infection rate was 7%, while the pulmonary and cardiac complication rates were 18% (19% vs. 17%, *p 0.857*) and 7% (7% vs. 6%, *p 0.838*), respectively. There was one patient in the neoadjuvant group who had postoperative acute kidney injury, warranting hemodialysis.

The reoperation rate was 13%, higher in the neoadjuvant group than the outright surgery (17% vs. 12%, *p 0.595*). The average length of hospital stay was 23 days ± 14, higher in the outright surgery than the neoadjuvant group (28 days ± 16.7 vs. 21 days ± 8.4, p 0.078). The in-hospital mortality rate was 7%, higher in the neoadjuvant group (11% vs. 5%, *p 0.353*). (Table 7)

**Table 7:** Postoperative outcomes of patients who underwent definitive surgery

Mean (SD) / Frequency (%)	Overall N = 61	Outright Definitive Surgery N = 43	Post-neoadjuvant therapy N = 18	*P* value
Surgical Complications				
Anastomotic leak	6 (10%)	4 (9%)	2 (11%)	.829
Chyle leak	1 (2%)	0	1 (6%)	.119
Surgical site infection	4 (7%)	3 (7%)	1 (6%)	.838
Medical Complications				
Pulmonary	11 (18%)	8 (19%)	3 (17%)	.857
Cardiac	4 (7%)	3 (7%)	1 (6%)	.838
Renal	1 (2%)	0	1 (6%)	.119
Length of Hospital Stay (days)	23 (SD 14)	28 (SD 16.7)	21 (SD 8.4)	.078
Re-operation	8 (13%)	5 (12%)	3 (17%)	.595
Anastomotic leak	5 (62%)	3 (60%)	2 (67%)	
Chyle leak	1 (13%)	0	1 (33%)	
Pleural effusion	2 (25%)	2 (40%)	0	
In-hospital Surgical Morbidity Rate	11 (18%)	7 (16%)	4 (22%)	
In-hospital Mortality Rate	4 (7%)	2 (5%)	2 (11%)	

### Adjuvant therapy

Data relating to adjuvant treatment was not available in all cases. There were nine who underwent adjuvant chemotherapy, five in the outright surgery group and four in the neoadjuvant group. Patients in the outright surgery group who underwent adjuvant chemotherapy were operated before 2020, but details on the regimen given were not recorded. Patients in the neoadjuvant group were given perioperative FLOT regimen (4 neoadjuvant cycles and 4 adjuvant cycles) and were managed 2022 onwards. There was no patient who received adjuvant chemoradiotherapy. (Table 8)

**Table 8:** Adjuvant therapy given after definitive surgery

Mean (SD) / Frequency (%)	Overall N = 61	Outright Definitive Surgery N = 43	Post-neoadjuvant therapy N = 18
Adjuvant chemotherapy			
• Fluorouracil, Leucovorin, Oxaliplatin and Docetaxel	4 (7%)	0	4 (22%)
• Not specified	5 (8%)	5 (12%)	0
Adjuvant chemoradiotherapy	0	0	0
Radiotherapy only	0	0	0
Targeted or immunotherapy	0	0	0
No adjuvant therapy given	52	38	14

### Long-term outcomes

The median follow-up was 55 months. Locoregional recurrence was observed in two patients within two years. One patient had Siewert 2 GEJ cancer with inadequate nodal harvest (2 positive lymph nodes out of 6) but adjuvant chemotherapy was given. The other patient had Siewert 3 classification with positive proximal margin and multiple positive lymph nodes (21 out of 40 lymph nodes). However, this patient did not receive adjuvant treatment. There were 19 patients who developed distant metastasis and majority them had no adjuvant treatment (10 of 12, 83%). (Table 9)

**Table 9:** Long-term follow-up of patients with GEJ carcinoma who underwent definitive surgery

Mean (SD) / Frequency (%)	Overall N = 61	Outright Definitive Surgery N = 43		Neoadjuvant Treatment N = 18	
		No adjuvant treatment N = 39	Adjuvant treatment N = 4	Neoadjuvant chemotherapy N = 13	Neoadjuvant chemoRT N = 5
2 year FOLLOW-UP					
Recurrence					
Locoregional	2 (3%)	1 (3%)	1 (25%)	0	0
Distant	16 (26%)	10 (26%)	2 (50%)	1 (8%)	3 (40%)
Death	43 (70%)	27 (64%)	4 (100%)	8 (54%)	4 (60%)
Cause of death					
Cancer-related	24 (57%)	16 (59%)	3 (75%)	3 (43%)	2 (50%)
Other cause	18 (43%)	11 (41%)	1 (25%)	4 (57%)	2 (50%)
5 year FOLLOW-UP					
Recurrence					
Locoregional	0	0	0	0	0
Distant	4 (6%)	3 (8%)	1 (25%)	0	0
Death	6 (10%)	4 (10%)	1 (25%)	0	1 (20%)
Cause of death					
Cancer-related	6 (100%)	4 (100%)	1 (100%)	0	1 (100%)
Other cause	0	0	0	0	0

The overall survival was determined with the help of the Philippine Statistics Authority (PSA) to gather data on the cause of death of the patients included in the study.

**Fig. 1:**
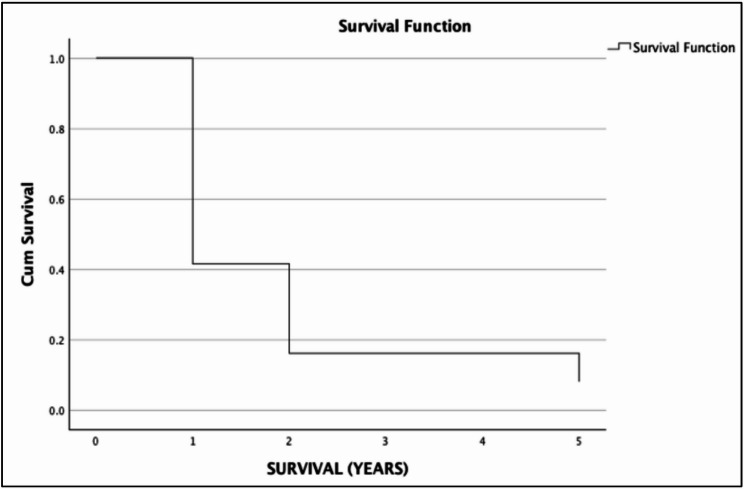
Overall survival of nonmetastatic GEJ cancer surgically managed in UP-PGH

**Fig. 2:**
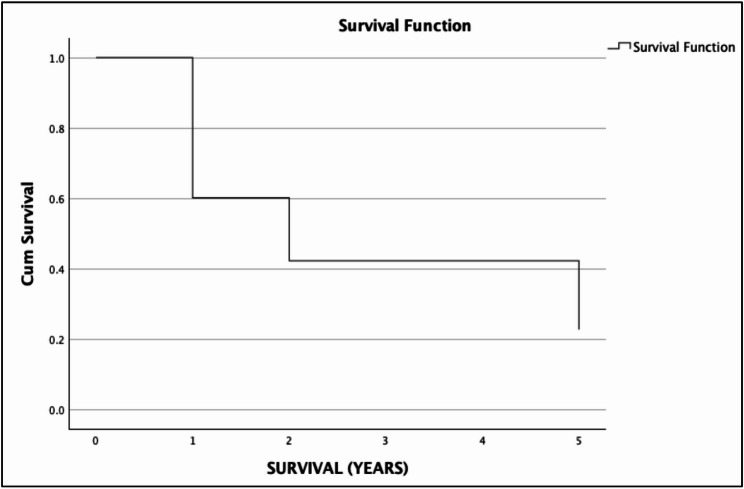
Cancer-specific survival of nonmetastatic GEJ cancer surgically managed in UP-PGH

**Fig. 3:**
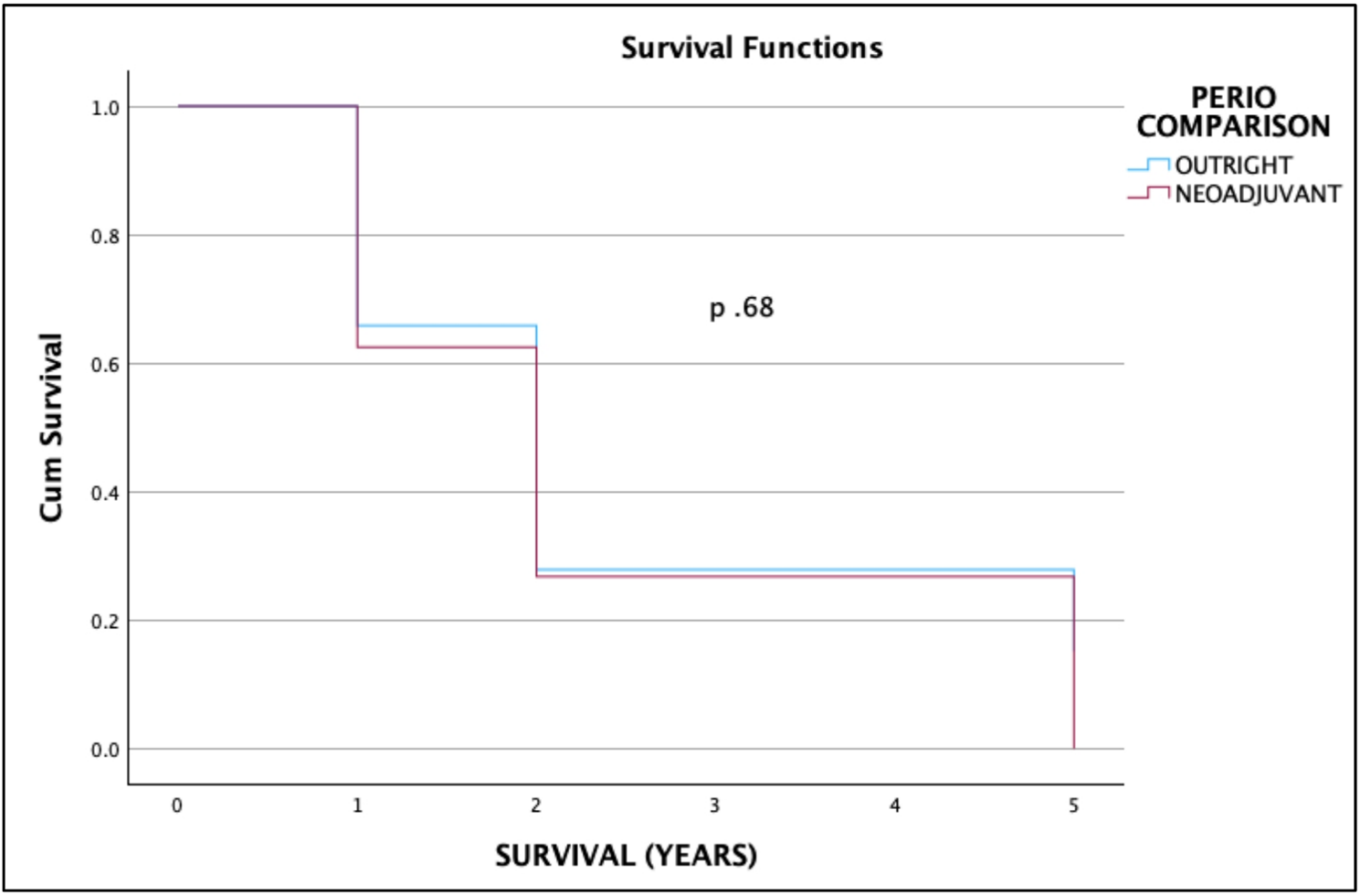
Overall survival of nonmetastatic GEJ cancer patients who underwent definitive surgery

**Fig. 4:**
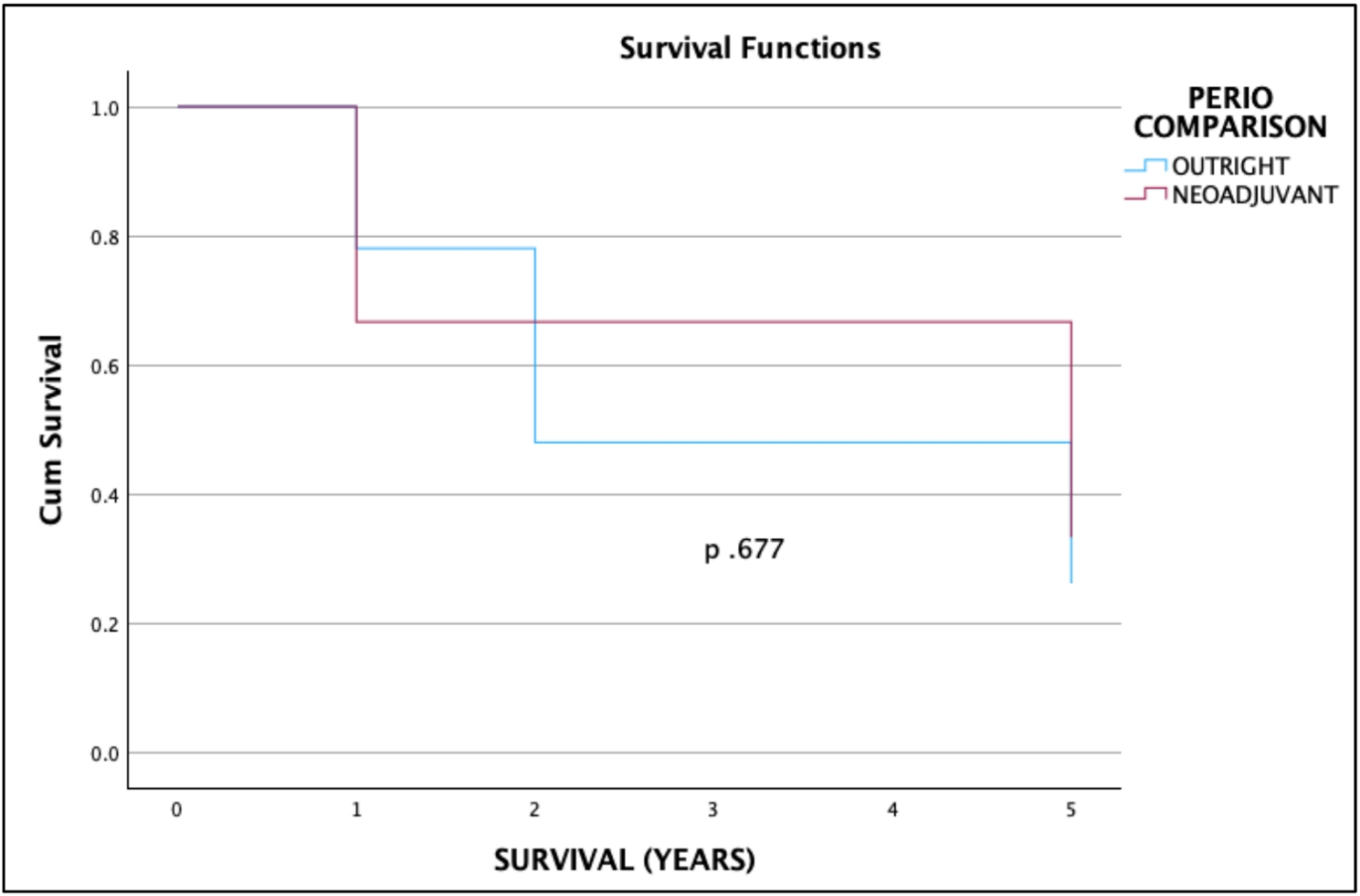
Cancer-specific survival of nonmetastatic GEJ patients who underwent definitive surgery

The 2- and 5-year overall survival were 14.75% (95% CI 8.88–24.22%) and 8.05% (95% CI 3.87–16.74%), respectively. The 2- and 5-year cancer-specific survival were 49.46% (95% CI 37.15–65.85%) and 26.98% (95% CI 14.65–49.69%), respectively. Notably, patients who received neoadjuvant treatment had improved cancer-specific survival with 2-year CSS of 51.85%, 95% CI 30.71–87.55% in the neoadjuvant group versus 48.66%, 95% CI 34.61–68.4% in the outright surgery group, and 5-year CSS of 51.85%, 95% CI 30.71–87.55% in the neoadjuvant group versus 26.54%, 95% CU 14.02–50.23% in the outright surgery group. However, there was no significant difference in the OS and CSS rates between the treatment modalities. (Figures 1, 2, 3 and 4)

## Discussion

This study provides significant insights into the management of non-metastatic gastroesophageal cancer in a resource-limited country like the Philippines.

The predominance of adenocarcinoma (85%) aligns with the global data indicating a rising incidence of GEJ adenocarcinoma in the Western and Asian populations. In the SEER database, adenocarcinoma predominates in the GEJ region, especially in the male population and in association with Barrett’s esophagus and obesity [7, 8]. The Siewert classification determined the management approach. The predominance of Siewert II and III cancers required multimodal treatment as emphasized in the NCCN guidelines. Also the predominance of Siewert III among patients who underwent outright surgery has been due to accessibility as total gastrectomy being the main treatment approach for tumors located distal to the GE junction [9, 10]. 

Margin positivity (10% proximal margin positivity, 3% distal margin positivity) is similar to the National Cancer Database (NCDB) analysis with 9.37% margin positivity. Neoadjuvant therapy has been associated with 63% reduced odds in margin positivity [11]. Notably, all of the patients who had positive margins did not undergo neoadjuvant therapy. Also, hospital and surgeon volume has been identified as modifiable risk factor for margin positivity following esophagectomy. A prior NCDB analysis categorized high-volume academic institutions as having > 13 cases/year [12]. The Leapfrog group has also recommended a minimum of 20 esophagectomies per year to reduce morbidity and mortality [13]. In this study, an average of 6 esophagectomies per year has been identified, which may also reflect the institution’s experience for the surgical procedure.

Neoadjuvant therapy has been underutilized in the earlier periods of the study. However, it has shifted with increase adoption reflecting adherence to international standards [14, 15]. FLOT regimen was most commonly used and it demonstrated superiority in outcomes, similar to the results of the FLOT4 trial [4]. However, 22% of the patients were unable to complete treatment due to treatment-related toxicity and disease progression, thus highlighting the importance of optimized patient selection and tailored neoadjuvant strategies. Similarly, chemoradiation using the CROSS regimen has demonstrated promise especially for locally advanced disease. In this study, neoadjuvant therapy had reduced margin positivity and tumor regression rates. The pCR rate of 6% achieved after neoadjuvant FLOT was similar to the results of meta-analysis in 2024, wherein the pooled prevalence of PCR was 6% (95% CI: 1%−12%) after neoadjuvant chemotherapy [16]. 

The 6% overall anastomotic leak rate is within the international published leak rates of 5–30% [17]. Similar to the ACS-NSQIP database analysis, patients who received neoadjuvant therapy and outright surgery had similar 30-day overall and serious morbidity, and length of hospital stay [18]. The in-hospital mortality rate of 7% is higher than published mortality rate in the ACS-NSQIP database analysis, reflecting institutional experience and the need for improvement in perioperative care protocols.

Although the CSS rate was higher in the neoadjuvant therapy group (51.85%, 95% CI 30.71–87.55%), the 2- and 5-year overall survival in the study remains poor (14% and 8%, respectively). This CSS rates were similar to a previous study in the same institution with 1-year disease-free survival of 69.4% [6]. These results emphasized the aggressive nature of GEJ cancers and the importance of more effective systemic therapies, including targeted therapies and immunotherapy which were not available in the institution.

The retrospective nature with incomplete medical records and poor follow-up limit its conclusions. Since data on adjuvant therapy and long-term follow-up in this study is sparse, comprehensive evaluation on recurrence patterns and survival outcomes has been limited. Also, the low-volume nature of the institution highlights the importance of the need to improve referral networks and collaboration to improve patient outcomes.

Based on these limitations, a prospective cohort study may be conducted in the future to assess improvements in the outcomes of the patients with adequate long-term follow-up data.

## Conclusions

This study presents the institutional experience of multimodal treatment strategies for non-metastatic GEJ cancer in a low-resource setting like the Philippines, including the increased adherence to international treatment guidelines to optimize perioperative therapy, minimizing complications and improving long-term outcomes. Strengthening institutional capabilities with multidisciplinary collaboration and advanced surgical oncology training along with improved access to current multimodal therapeutic options will be pivotal in addressing these gaps.

## Data Availability

The datasets generated or analyzed in this study are available from the corresponding author upon reasonable request.
